# Dense-TNT: Efficient Vehicle Type Classification Neural Network Using Satellite Imagery

**DOI:** 10.3390/s24237662

**Published:** 2024-11-29

**Authors:** Ruikang Luo, Yaofeng Song, Longfei Ye, Rong Su

**Affiliations:** School of Electrical and Electronic Engineering, Nanyang Technological University, Singapore 639798, Singapore; ruikang001@e.ntu.edu.sg (R.L.); yaofeng.song@ntu.edu.sg (Y.S.); longfei001@e.ntu.edu.sg (L.Y.)

**Keywords:** deep learning, transformer, remote sensing, vehicle classification

## Abstract

Accurate vehicle type classification plays a significant role in intelligent transportation systems. It is critical to understand the road conditions and usually contributive for the traffic light control system to respond correspondingly to alleviate traffic congestion. New technologies and comprehensive data sources, such as aerial photos and remote sensing data, provide richer and higher-dimensional information. In addition, due to the rapid development of deep neural network technology, image-based vehicle classification methods can better extract underlying objective features when processing data. Recently, several deep learning models have been proposed to solve this problem. However, traditional purely convolution-based approaches have constraints on global information extraction, and complex environments such as bad weather seriously limit their recognition capability. To improve vehicle type classification capability under complex environments, this study proposes a novel Densely Connected Convolutional Transformer-in-Transformer Neural Network (Dense-TNT) framework for vehicle type classification by stacking Densely Connected Convolutional Network (DenseNet) and Transformer-in-Transformer (TNT) layers. Vehicle data for three regions under four different weather conditions were deployed to evaluate the recognition capability. Our experimental findings validate the recognition ability of the proposed vehicle classification model, showing little decay even under heavy fog.

## 1. Introduction

**Figure 1:**
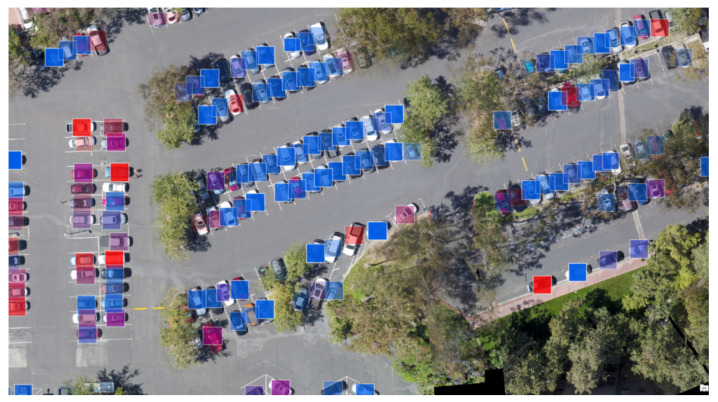
Deep learning algorithms can capture huge amounts of vehicle information in a specific region based on remote sensing data.

Vehicle type classification is one of the most important parts of an intelligent traffic system. Vehicle classification results can contribute to traffic parameters statistics, regional traffic demand and supply analysis, time series traffic information prediction [1], and transportation facilities usage management [2,3]. Example of remote sensing data vehicle classification is shown in Figure 1. In combination with appropriate data processing techniques such as missing data imputation and map matching, this can provide further traffic management guidance [4]. Traditional vehicle type classification methods are mainly based on sensor feedback such as magnetic induction and ultrasonic data [5,6]. Thanks to the extensive use of UAV surveillance and satellite remote sensing data, image-based solutions towards intelligent traffic system are being rapidly developed. Image processing approaches can be divided into appearance-based methods and deep learning-based methods. Appearance-based methods usually generate a 3D parameter model to represent the vehicle for classification, while deep learning-based methods apply image recognition algorithms to extract objective features that can be used to classify vehicles.

Although remarkable efforts have been made in remote sensing classification, these methods are not ideal when applied to real situations. There are three main limitations on processing remote sensing data. First, high-resolution satellite remote sensing images are expensive, and most accessible open-source datasets are in low-resolution [7]. The poor quality of these images constrains model performance. Second, optical remote sensing images are highly affected by weather conditions [8]. Complex weather conditions such as fog and haze lead to degraded and blurred images. Third, some modern progressive car designs make distinction boundaries ambiguous. It is essential to determine vehicle types by considering both local and global dependencies, which places higher requirements on the model design process.

To overcome these issues, existing studies have provided solutions in two directions. The first line of work focuses on haze removal or visibility enhancement by utilizing methods such as Image Super-Resolution (ISR) [9] to sharpen edges and further improve resolution. Several methods adopt denoising operations to remove haze and obtain clearer processed images [10]. However, due to the pixel degradation caused by inherent statistical features of fog and haze, these removal methods may not be effective for processing satellite remote sensing images. The other direction of work involves the construction of new end-to-end deep learning algorithms. However, as stated above, it is quite challenging to capture both local and global information under comprehensive conditions.

This study proposes a novel Dense-TNT model for all-weather vehicle classification. The proposed model combines a DenseNet layer with a TNT layer. This latter type of layer based on the general transformer architecture was recently introduced for objective detection as a way to more effectively extract image features. The proposed model is evaluated using a real-world satellite dataset under various weather conditions by classifying objects into three categories: sedan, pickup, and other. Sedans and pickups are the two primary types of vehicles on the road, and obtaining accurate distribution information for these three vehicle categories provides significant benefits to intelligent transportation systems in terms of several different aspects. Classification results can be applied in areas such as multimodal traffic characteristic prediction, Estimated Time of Arrival (ETA) prediction, and smart traffic management. In summary, this paper makes three main contributions:

- A novel Dense-TNT model containing a DenseNet layer and TNT layer for vehicle type recognition. The proposed method has better ability to understand the global pattern of objectives based on the existing knowledge.
- Extensive analysis regarding vehicle type classification over remote sensing images collected from three different regions. This analysis validates the proposed model based on its superior recognition capability compared to several baseline models.
- We use data from three real-world regions under normal weather condition, with appropriate filters added to simulate light, medium, and heavy haze conditions. The evaluation results show around 80% classification accuracy even under heavy haze, with an improvement in accuracy of around 5–10% over the baseline algorithms. These results verify the feasibility of the proposed method.

The rest of this paper is organized as follows: in Section 2, recent research on vehicle classification is introduced; in Section 3, the proposed Dense-TNT framework is described in detail; Section 4 describes the experimental settings and evaluates the vehicle recognition performance in comparison to baseline models using different datasets and weather conditions; finally, Section 5 provides our conclusions and future research plans.

## 2. Related Work

Existing vehicle type classification methods can be divided into three categories: appearance-driven methods [11,12,13,14,15], model-based methods [16,17,18], and deep learning-based methods. Appearance-driven methods focus on extracting vehicle appearance features, then try to classify vehicle types by comparing these features with known vehicle features. In [19], the authors proposed a method for extracting distinctive invariant features and performed robust matching within a known database based on the indicated probability. In this approach, the quantity and quality of known data largely determine the classification performance, and it is difficult for the model to provide accurate recognition when the features of the target object are not in the database collection. Unfortunately this is a common scenario, as vehicle designs can differ widely. Model-based methods focus on computing vehicles’ 3D parameters and recovering a 3D model used for classification. In [20], a parameterized framework was designed to represent a single vehicle with twelve shape parameters and three pose parameters. The local gradient-based method was applied to evaluate the goodness of fit between the vehicle projection and known data. However, similar to appearance-driven methods, the appearance and dimension of vehicles can be disturbed and degraded by poor data collection and complex weather condition [21]. Moreover, both model-based and appearance-driven methods rely heavily on substantial prior knowledge about the classification objectives, which can be challenging to obtain in many real-world applications. Thus, in this study we mainly discuss deep learning-based methods, which have the ability to capture more information.

Among deep learning-based methods, Convolutional Neural Networks (CNNs) and variants thereof play a significant role in existing image processing approaches [22]. In the classical CNN structure, convolution layers and pooling layers are stacked, allowing CNNs to automatically learn multistage invariant features for specific objects via trainable kernels [23]. A CNN takes a vehicle images as input and generates each vehicle type probability. However, the pooling operation means that CNNs can ignore some valuable information due to the absence of careful screening of the correlation between the parts and the entire object [24]. Thus, there has been a great deal of interest in combining convolutional layers with attention mechanisms for image classification tasks to address the unbalanced importance distribution over a single image [25]. To increase the interpretability of CNNs, some research has applied semi-supervised learning by using unlabeled data in pretraining process and learning output parameters in a supervised way [26].

The transformer architecture was first proposed in 2017 for natural language processing (NLP) tasks. The principle of the attention mechanism leads to quadratic computational costs when directly applying the transformer architecture to image processing, as each pixel needs to attend to every other pixel. Therefore, in order to adopt transformer-like structures for image processing, adaptive adjustments are necessary. In [27], self-attention was applied in local neighborhoods to save operations and replace convolutions [28]. Sparse Transformer [29] uses a scalable filter to adjust the global self-attention before processing images, while the recent MetaFormer [30] replaces the attention mechanism with a token mixer while retaining the general transformer architecture. Even when simply introducing a pooling layer inside a token mixer has been found to lead to superior performance. Although CNNs are the fundamental model in vision applications, transformers have a great potential to provide an alternative approach.

Vision Transformer (ViT) has been widely used and verified to be efficient in many scenarios, including object detection, segmentation, pose estimation, image enhancement, and video captioning [31]. The canonical ViT structure divides one image into sequence patches and treats each patch as one input element for classification. Due to the inherent characteristics of transformers, ViT is good at long-range relationship extraction but poor at capturing local features, as 2D patches are compressed into a 1D vector. Thus, previous works have tried to improve the local modeling ability [32,33,34] by introducing extra architectures to model the inner correlation patch-by-patch and layer-by-layer [35,36]. In [34], the authors proposed a hybrid token generation mechanism to obtain local and global information from regional tokens and local tokens. In addition to efforts around enhancing the local information extraction capability of ViT, other directions include improving the self-attention calculation [37], encoding [38,39], and normalization strategy [40]. In [30], the authors achieved qualified performance by simplifying the structure even without an attention mechanism.

Due to the need to use low-resolution data sourced from satellite imagery and the existence of complex real-world noise, local and global information extraction are both significant challenges for accurate vehicle classification. Even though ViT tends to focus on low-resolution features due to repeated downsampling processes, loss of fine-grained localization information occurs during feature extraction, making ViT unsuitable for low-level image recognition tasks [41]. Meanwhile, unlike CNNs, which inherently build hierarchical feature representations, ViT lacks this inductive bias, making it less effective at capturing the local features which are crucial for low-resolution images. Instead of embedding a nesting structure within the transformer, in this study we stack suitable CNN and ViT variants to construct a novel efficient architecture for vehicle classification task while avoiding the need for complex computation. By integrating convolutional layers into the feature extraction process, more localized information can be extracted prior to downsampling. This approach is expected to achieve satisfying recognition performance even under complex conditions. If realized, this technology could even be deployed on nanosatellites for other recognition tasks [42].

## 3. Methodology

### 3.1. Problem Analysis

The main purpose of this paper is to build a novel end-to-end vehicle classification model combining selected CNN and ViT variants. The proposed model uses satellite remote sensing images of various vehicle types from different regions under different weather conditions as input to perform image processing and generate vehicle type classification results. The principle and detailed architecture of the proposed model is illustrated in this section.

### 3.2. Transformer Layer: TNT

ViT has been successfully applied for a wide range of scenarios, and has proven to be efficient thanks to its ability to extract global long sequence dependencies; however, in terms of local information aggregation performance there is still a gap between ViT and CNNs. Even though some works have proposed variants that enhance ViT’s local extraction ability, combination with CNNs is a more direct method to equip transformer architectures with local capture ability.

Similarly, after a careful review of the literature, TNT [32] was selected as the variant in our proposed hybrid model. In the canonical ViT structure, input images are divided into long sequence patches without local correlation information, which makes it difficult for transformers to capture the relationship simply based on the 2D patch sequence. Compared to ViT, the main advantages of TNT in terms of the current study are its introduction of a fragmentation mechanism to create sub-patches within every patch. As the name indicates, the TNT architecture contains one internal transformer that models the correlation between sub-patches and another external transformer that propagates information among patches. If the *n*-length patch sequence $X^{i}=\left[X^{1},X^{2},\cdots ,X^{n}\right]$ is regarded as a visual sentence, each sentence is further divided into *m* visual words for embedding:

(1) $$X^{i}\overset{embedding}{\to }Y^{i}=\left[y^{i,1},y^{i,2},\cdots ,y^{i,m}\right].$$

For internal transformers, the data flow can be expressed as

(2) $${Y^{\prime }}_{l}^{i}=Y_{l-1}^{i}+MSA\left(LN\left(Y_{l-1}^{i}\right)\right),$$

(3) $$Y_{l}^{i}={Y^{\prime }}_{l}^{i}+MLP\left(LN\left({Y^{\prime }}_{l}^{i}\right)\right),$$

where *l* is the index of visual words, MSA means multihead self-attention, MLP means multi-layer perceptron, and LN means layer normalization. Thus, the overall internal transformations are

(4) $$Y_{l}=\left[Y_{l}^{1},Y_{l}^{2},\cdots ,Y_{l}^{n}\right].$$

Further, the sequence is transformed from words

(5) $$Z_{l-1}^{i}=Z_{l-1}^{i}+FC\left(Vec\left(Y_{l}^{i}\right)\right),$$

where FC refers to the fully connected layer. Then, the entire sentence embedding sequence is represented as $Z_{0}=\left[Z_{class},Z_{0}^{1},Z_{0}^{2},\cdots ,Z_{0}^{n}\right]$, where $Z_{class}$ is the class token. Finally, the data flow of the external transformer is formulated as

(6) $${Z^{\prime }}_{l}=Z_{l-1}+MSA\left(LN\left(Z_{l-1}\right)\right),$$

(7) $$Z_{l}={Z^{\prime }}_{l}+MLP\left(LN\left({Z^{\prime }}_{l}\right)\right).$$

**Figure 2:**
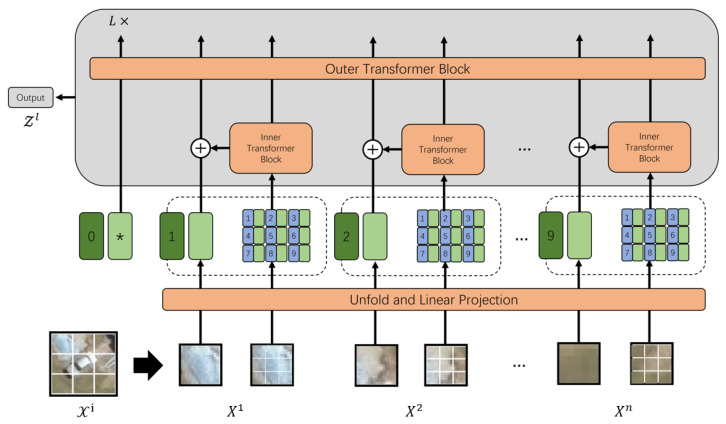
Illustration of TNT model details. Apart from positional embeddings, Mark * in the figure refers to other learnable embeddings.

In the original paper, the authors showed better classification performance of TNT compared to several baselines, including ViT. The TNT architecture is shown in Figure 2.

### 3.3. Convolutional Layer: DenseNet

As discussed in the previous sections, CNNs usually show better local fixed information extraction capability thanks to their use of a kernel structure and convolution operations [43]. Thus, the convolutional layer was retained when designing our efficient image recognition model. DenseNet is designed for localized spatial information extraction to overcome the problem of losing fine-grained localization information; thus, we chose DenseNet as the locality information extractor in this research. Compared to other commonly used CNN models such as ResNet and GoogLenet, DenseNet connects each convolutional layer with every other layer using the feed-forward network instead of through sequential connections between layers, as in ResNet and other models. This approach is called dense connectivity [44]. The *i*-th layer is formulated as Equation (8):

(8) $$Z_{i}^{*}=H_{i}\left(\left[Z_{0},Z_{1},\ldots ,Z_{i-1}\right]\right)$$

where $\left[Z_{0},Z_{1},\cdots ,Z_{i-1}\right]$ is the concatenation result of the feature maps from all previous layers and $H_{i}\left(\cdot \right)$ is the composite function combining the batch normalization, rectified linear unit, and convolution operations. The convolutional layers initially extract features from the input data, which are subsequently passed through the ReLU activation function. The ReLU outputs are then normalized to enhance training stability and performance.

**Figure 3:**
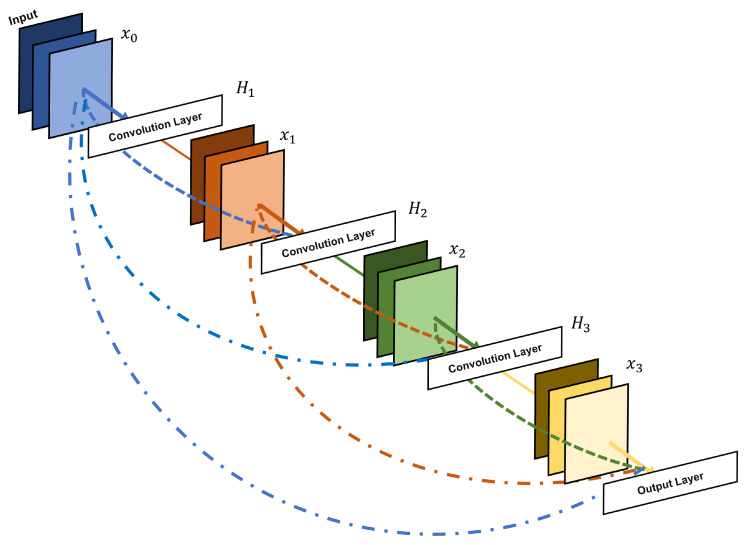
DenseNet model structure showing a four-layer dense block. Each layer takes all preceding feature maps as input. The convolutional layers between two adjacent blocks are used to adjust the size of the feature map.

In this case, every layer takes the feature maps from all preceding convolutional layers. This enhances the information propagation capability to avoid the issue of serious dependencies loss from distant stages, and also helps to alleviate the vanishing gradient problem. Due to the complex environment of vehicle remote sensing imagery, such as hazy weather conditions and shadowed regions, this enhanced feature extraction capability is exactly what we need during training. An illustration of the DenseNet layout is shown in Figure 3.

### 3.4. Classifier Layer

To complete the vehicle classification task, the probability of each type is expected to be calculated based on the output feature maps from previous layers. Thus, the softmax classifier layer is added as the final part of our proposed model to take the output feature vector from TNT layer and generate vehicle type probability vector for the choice with highest probability. The learnable linear function modeling the relationship can be expressed as follows:

(9) $$v=W^{T}Z^{*}+b$$

where $Z^{*}\in R^{D\times 1}$ is the real number output feature with dimension *D* from TNT, *W* is the parameter to be learned, $v\in R^{C\times 1}$ is the vehicle type variable, *C* is the number of vehicle types, and *b* is the bias of the linear mapping. To emphasize the vehicle type with the highest probability, softmax is applied to achieve the final normalized output $O={\left[O_{1},O_{2},\cdots ,O_{C}\right]}^{T}$. For the *i*-th class prediction $v_{i}$, the final output $O_{i}$ can be calculated as

(10) $$V=\sum_{i=1}^{C}e^{v_{i}},$$

(11) $$O_{i}=\frac{1}{V}e^{v_{i}}.$$

### 3.5. Dense-TNT Overview Model

**Figure 4:**
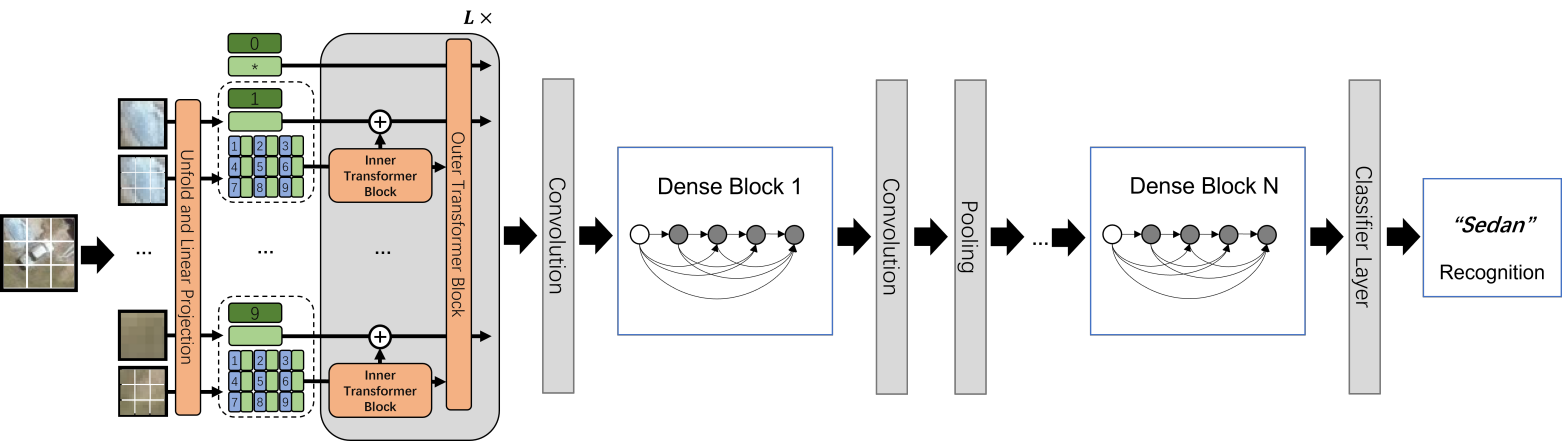
The architecture of the proposed Dense-TNT neural network, consisting of TNT and DenseNet parts. The classifier layer serves as the recognition layer used to compute the type probability of the input vehicle. Mark * in the figure refers to other learnable embeddings.

In summary, our novel Dense-TNT model is designed based on DenseNet and TNT as shown in Figure 4. It contains two parts: (1) the transformer-based layer, which guarantees baseline reasonable performance; and (2) the convolutional layer, which captures local fixed features. DenseNet is beneficial for its kernel and convolutional operation, widely used for image recognition, and has deeper locality extraction capability than other CNN variants, while TNT is adept at global information capture and provides better understanding than canonical ViT. The proposed Dense-TNT model can processes the information propagated through this hybrid structure by extracting some specific local features, further improving the recognition capability even under complex environmental conditions such as haze and fog.

## 4. Experiments

To evaluate our Dense-TNT model, we compared its classification performance with that of several advanced baselines, including PoolFormer and ViT. The main classification task is to classify sedans and pickups from pictures taken by remote sensors from three different areas. To comprehensively evaluate the model’s performance in multiclass classification, we further simulated satellite remote sensing using a drone remote sensing dataset and conducted a seven-class classification task. In addition, we evaluated the classification ability of Dense-TNT in a two-class classification scenario when the input pictures were affected by fog and darkness.

### 4.1. Evaluation Criteria

To evaluate classification outcomes for all models, four criteria were applied to assess the performance results. In the case of two-class classification:

- True Positive (TP): A sedan is successfully recognized as a sedan.
- True Negative (TN): A pickup is successfully recognized as a pickup.
- False Positive (FP): A pickup is successfully recognized as a sedan.
- False Negative (FN): A sedan is successfully recognized as a pickup.

Thus, the following four criteria are formulated:

(12) $$Accuracy=\frac{TP+TN}{TP+TN+FP+FN},$$

(13) $$Precision=\frac{TP}{TP+FP},$$

(14) $$Recall=\frac{TP}{TP+FN},$$

(15) $$F1-score=\frac{2\times Precision\times Recall}{Precision+Recall}.$$

The higher the evaluation score, the better the recognition performance.

In a multiclass classification task with *C* classes, the following terms are defined for each class i∈{1,2,⋯,C}:

- True Positive (*TP_i_*): The number of instances correctly predicted as belonging to class *i*:
  (16) $$TP_{i}=M_{ii}$$
  where $M_{ii}$ is the diagonal element of the confusion matrix, representing instances where both the actual and predicted class are *i*.
- False Positive (*FP_i_*): The number of instances incorrectly predicted as class *i* when the actual class is not *i*:
  (17) $$FP_{i}=\sum_{j=1,j\neq i}^{C}M_{ji}$$
  where $M_{ji}$ represents the instances actually belonging to class *j* but predicted as class *i*.
- False Negative (*FN_i_*): The number of instances of class *i* incorrectly predicted as another class:
  (18) $$FN_{i}=\sum_{j=1,j\neq i}^{C}M_{ij}$$
  where $M_{ij}$ represents the instances actually belonging to class *i* but predicted as class *j*.
- True Negative (*TN_i_*): The number of instances correctly predicted as not belonging to class *i*, that is, the total number of instances minus those involved in $TP_{i}$, $FP_{i}$, and $FN_{i}$:
  (19) $$TN_{i}=\sum_{k=1}^{C}\sum_{l=1}^{C}M_{kl}-\left(TP_{i}+FP_{i}+FN_{i}\right)$$
  where $M_{kl}$ represents all elements of the confusion matrix.

The confusion matrix for *C*-class classification is represented as a C×C matrix:

$$\left[\begin{array}{cccc} M_{11} & M_{12} & \cdots & M_{1C}\\ M_{21} & M_{22} & \cdots & M_{2C}\\ \vdots & \vdots & \ddots & \vdots \\ M_{C1} & M_{C2} & \cdots & M_{CC} \end{array}\right]$$

where:

- $M_{ij}$ is the number of instances with actual class *i* and predicted class *j*.
- Diagonal elements ($M_{ii}$) represent correct predictions (True Positives for class *i*).
- Off-diagonal elements ($M_{ij}$ for i≠j) represent misclassifications.

Thus, the four criteria for multiclass classification are formulated as follows:    

(20) $$Accuracy=\frac{\sum_{i=1}^{C}TP_{i}}{\sum_{i=1}^{C}\left(TP_{i}+FP_{i}+FN_{i}\right)},$$

(21) $$Precision_{i}=\frac{TP_{i}}{TP_{i}+FP_{i}},$$

(22) $$Recall_{i}=\frac{TP_{i}}{TP_{i}+FN_{i}},$$

(23) $$F1-score_{i}=\frac{2\times Precision_{i}\times Recall_{i}}{Precision_{i}+Recall_{i}}.$$

### 4.2. Experiment Settings

All settings, including baseline models and training settings, were kept the same in the following experiments. The baseline models were PoolFormer and ViT. PoolFormer is the specific framework proposed in [30], and achieves strong recognition performance. ViT is widely applied in image processing problems, as discussed in Section 2. We applied Dense-TNT with parameter sizes of s12 and s24, PoolFormer with parameter sizes of s12 and s24, and ViT with two layers and twelve layers. The parameter settings of the baseline models and the Dense-TNT model are shown in Table 1. The dense blocks for the Dense-TNT S12 and Dense-TNT S24 models both consisted of five convolutional layers with 5 × 5 kernels and a stride of 1.

The models were trained over 50 epochs on an RTX3060 GPU with a maximum learning rate of $lr=2e^{-3}$. The AdamW optimizer was used with a weight decay of 0.05. The batch size was set to 0.01 of the training dataset. The size of the training dataset was 0.8 of the whole dataset, while the size of test dataset was 0.2 of the whole dataset. All the training and test data were randomly selected from the whole dataset. The number of different vehicle samples are kept consistent considering the balance of the number of different vehicle types. See Algorithm for the detailed training process (Algorithm 1).
**Algorithm 1:** Dense-TNT Model Training.
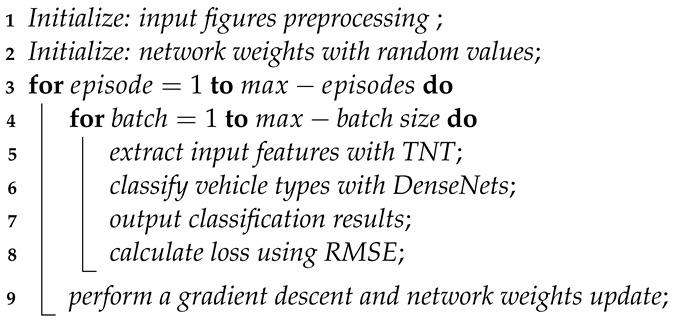


### 4.3. Classification in Normal Weather Conditions

#### 4.3.1. Data Description

We used Cars Overhead With Context (COWC accessed in 12 September 2024) (http://gdo-datasci.ucllnl.org/cowc/) [45], a remote sensing target detection dataset with a resolution of 15 cm per pixel and an image size of 64 × 64, to perform the classification. Remote sensing pictures from three different areas (Toronto, Canada; Selwyn New Zealand; and Columbus, Ohio USA) were selected. Details of the different area datasets are described in Table 2 and example pictures of sedans and pickups are shown in Table 3.

#### 4.3.2. Experiment Results and Analysis

**Figure 5:**
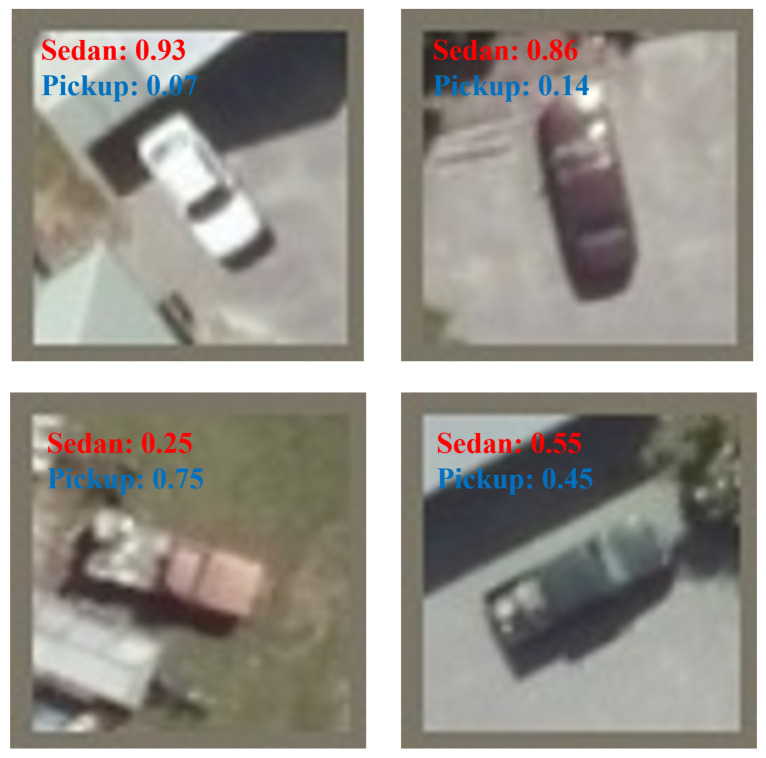
Classification results with corresponding probabilities under normal weather conditions.

Table 4 shows the experimental results. Figure 5 shows the classification results with probabilities after processing under the normal weather condition.

In comparing ViT l12, Dense-TNT s24, and PoolFormer s24, Dense-TNT achieves generally better performance on all datasets. Even though the computation costs of Dense-TNT s24 and PoolFormer s24 are both smaller than that of ViT l12, the accuracy results of both models are relatively higher. In the comparison between the smaller Dense-TNT s12, PoolFormer s12 and ViT l2 models, Dense-TNT again performs better than the others. Due to its larger amount of parameters, Dense-TNT s24 has relatively better performance than Dense-TNT s12.

**Figure 6:**
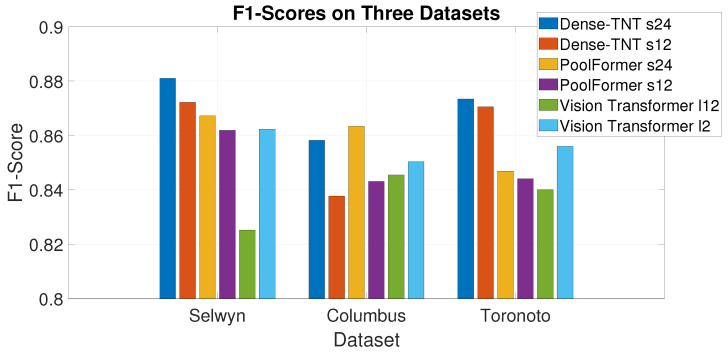
F1-scores for the three datasets under normal weather conditions.

The F1-score is the harmonic mean of the precision and recall, and reflects the robustness of a model’s recognition capability. Figure 6 shows the F1-scores in histogram form. It can be observed that Dense-TNT again has superior performance.

### 4.4. Multiclass Classification

#### 4.4.1. Data Description

**Figure 7:**
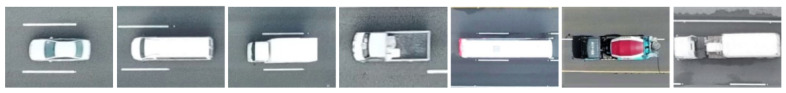
Example images from the VAID dataset; from left to right: sedan, minibus, truck, pickup truck, bus, cement truck, and trailer.

To comprehensively evaluate the proposed Dense-TNT model on a multiclass classification task, we conducted a seven-type vehicle classification experiment on a remote sensing dataset obtained via drone. The dataset for this experiment was the Vehicle Aerial Imaging from Drone (VAID accessed on 22 November 2024) dataset (https://vision.ee.ccu.edu.tw/aerialimage/) [46], which contains 6000 aerial images of different places in Taiwan taken under varying illumination conditions and from different viewing angles. This dataset includes multiple vehicle types captured by camera, including sedans, minibuses, trucks, pickups, buses, cement trucks, and trailers. Example images from the VAID dataset are shown in Figure 7.

#### 4.4.2. Experimental Results and Analysis

For this experiment, 500 samples were selected for each vehicle type to ensure a comprehensive and balanced analysis. Dense-TNT s24, Dense-TNT s12, PoolFormer s24, PoolFormer s12, ViT l12, and ViT l2 were again selected for model evaluation. We calculated the average precision, recall, and F1-score as evaluation criteria for all vehicle types. The classification results are shown in Table 5.

Table 5 presents the experimental results in the multiclass classification scenario. Compared to the results in Section 4.3, the models achieved relatively higher accuracy, precision, recall, and F1-scores, which is due to the input data having higher resolution and containing more spatial information. The proposed Dense-TNT s24 model outperforms the baseline models. These results demonstrate the effectiveness of combining convolutional feature extraction with the attention mechanism, which balances global attention across all input patches while preserving local detail.

**Figure 8:**
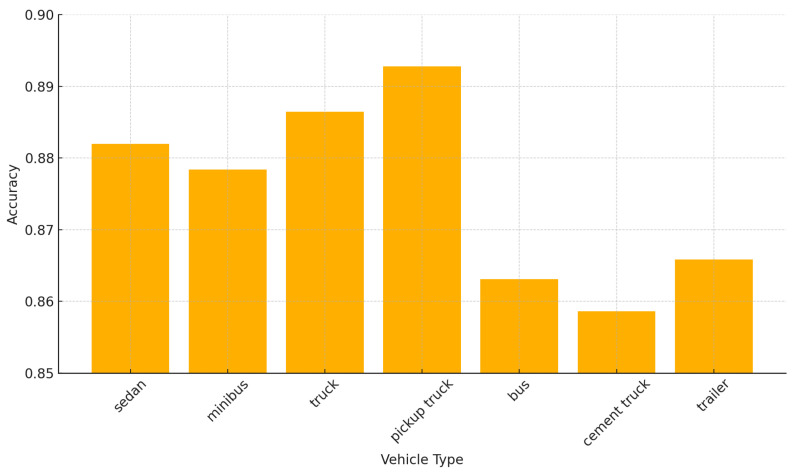
Classification accuracy for different vehicle types.

Figure 8 shows the classification accuracy of the Dense-TNT s24 model for all seven vehicle types. Dense-TNT demonstrates exceptional performance in vehicle classification, achieving high accuracy across all categories, with an average exceeding 87.5%. It performs particularly well on pickup trucks and sedans, reflecting its ability to effectively extract features for these classes. While cement trucks and buses have slightly lower accuracy, this could be attributed to their structural complexity and feature overlap with other categories. These results highlight the robustness and adaptability of Dense-TNT for multiclass vehicle recognition, with potential for further improvement through targeted data augmentation and feature optimization.

### 4.5. Classification in Foggy Condition

#### 4.5.1. Data Preprocessing

To obtain vehicle images under different fog conditions, we processed the grayscale value of every pixel in the vehicle image. Based on the grayscale value I(x,y) of the pixel on the *x*-th row and *y*-th column in the original image, new different grayscale values $I{\left(x,y\right)}^{\prime }$ were obtained based on the parameter β, as follows:

(24) $$d=-0.04\times \sqrt{{\left(x-x_{0}\right)}^{2}+{\left(y-y_{0}\right)}^{2}}+\sqrt{max(N,M)}$$

(25) $$t_{d}=e^{-\beta \times d}$$

(26) $$I{\left(x,y\right)}^{\prime }=I\left(x,y\right)\times t_{d}+0.5\times \left(1-t_{d}\right)$$

where $x_{0}$ and $y_{0}$ refer to the center of each row and column, respectively, *N* and *M* refer to the number of pixels in each row and column, respectively, *e* is the natural exponential base and parameter, and β can be adjusted to simulate varying levels of fog intensity. In the experiment, β was chosen as 0.08, 0.16, and 0.24 to realize three levels of foggy conditions, denoted light fog, medium fog, and heavy fog. The dataset collected in Selwyn, New Zealand was randomly chosen for the experiments. Table 6 shows example pictures under different levels of weather impact. The samples were selected from the COWC dataset and preprocessed to simulate foggy condition.

#### 4.5.2. Experimental Results and Analysis

**Figure 9:**
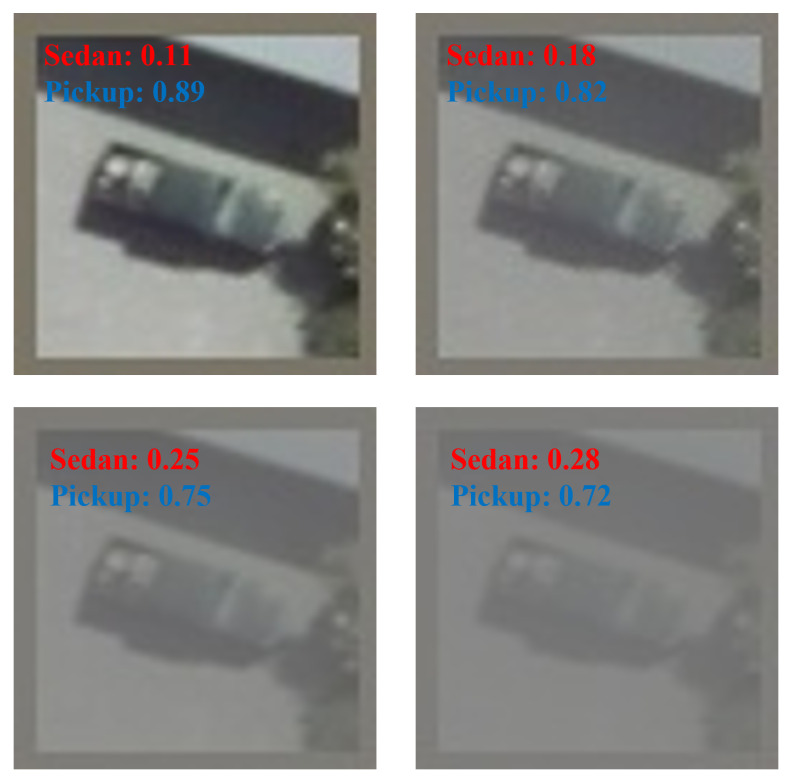
Classification results with corresponding probabilities under foggy weather conditions.

In this experiment, we kept the evaluation criteria the same as in Section 4.3 and used the same six models (Dense-TNT s24, Dense-TNT s12, PoolFormer s24, PoolFormer s12, ViT l12, and ViT l2). Table 7 shows the results of the experiment. Figure 9 shows the classification results with probabilities after processing under foggy weather conditions.

**Figure 10:**
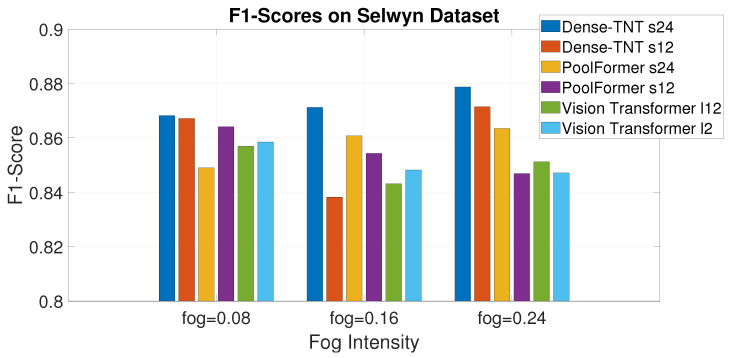
F1-scores on the Selwyn, New Zealand dataset under different levels of fog.

Figure 10 shows the F1-scores of the models in the experiment in the form of a histogram. Again, Dense-TNT has better performance than the baselines, with Dense-TNT s24 showing the best performance. Notably, this performance advantage increases as the fog becomes heavier, with the proposed model leading the baselines by even more.

When the input data are affected by different levels of fog, there is a certain level of decay in the accuracy of all six models. Despite this decrease in accuracy, Dense-TNT s24 still has a relatively better performance than PoolFormer s24 and ViT l12. Dense-TNT s12 also has generally better performance than PoolFormer s12 and ViT l2, demonstrating that Dense-TNT can still be useful when dealing with weather-affected input data.

### 4.6. Classification in Darkness Condition

#### 4.6.1. Data Preprocessing

We introduced a uniform darkness effect to the image dataset to simulate a dark environment. For every pixel in the image, the grayscale value was multiplied by a darkness factor θ∈ [0, 1]. In this case, the transformation of the grayscale value can be express as

(27) $$I{\left(x,y\right)}^{\prime }=I\left(x,y\right)\times \theta .$$

In this experiment, the value of the θ parameter was chosen as 0.8, 0.64, and 0.32 to realize three darkness conditions: light, medium, and heavy. The dataset collected in Selwyn, New Zealand was randomly chosen for this experiment. Table 8 shows example pictures under different levels of darkness impact.

#### 4.6.2. Experimental Results and Analysis

Table 9 presents the experimental results, showcasing the performance of various models under different darkness conditions. The results provide a comprehensive comparison of the models’ F1-scores across light, medium, and heavy darkness scenarios, highlighting their effectiveness and robustness under varying levels of visibility impairment.

The F1-score performance of the six models is presented under the following darkness conditions: light (θ = 0.8), medium (θ = 0.64), and heavy (θ = 0.32). Under the light darkness condition, Dense-TNT s24 exhibited the highest F1-score of 0.8912, demonstrating superior performance, followed closely by ViT l12 with an F1-score of 0.8776. The other models achieved F1-scores ranging from 0.8370 to 0.8783. Under medium darkness conditions, Dense-TNT s24 again showed outstanding performance, with the highest F1-score of 0.9048, while PoolFormer s12 ranked second with an F1-score of 0.8534 and the F1-scores of the other models ranged from 0.8264 to 0.8385. Under heavy darkness conditions, Dense-TNT s24 again performed the best, with an F1-score of 0.8761. ViT l12 and PoolFormer s24 followed with F1-scores of 0.8591 and 0.8295, respectively, and the other models achieved F1-scores ranging from 0.8225 to 0.8517. This summary highlights the consistent performance of Dense-TNT s24 across all darkness conditions, particularly excelling in the light and medium darkness scenarios.

**Figure 11:**
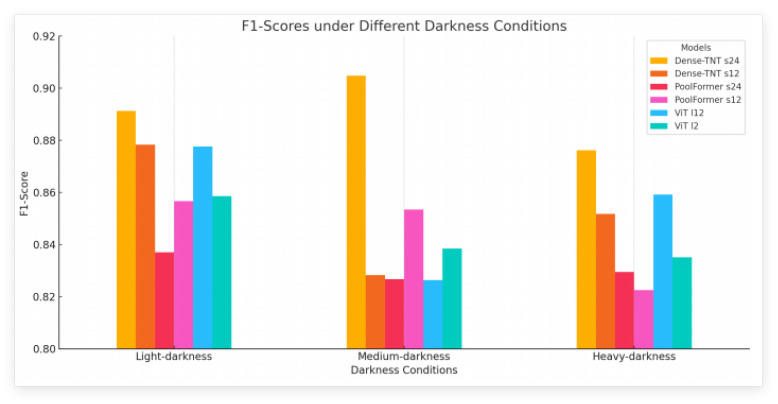
F1-scores on the Selwyn, New Zealand dataset under different darkness condition.

Figure 11 illustrates the F1-score performance of the six models across three different darkness conditions: light, medium, and heavy. Dense-TNT s24 consistently achieves the highest F1-scores in all conditions, indicating its superior adaptability and accuracy. ViT l12 also performs well, particularly under light and medium darkness conditions. The chart highlights how model performance can vary significantly with changes in environmental visibility, emphasizing the importance of selecting the right model for specific darkness conditions.

## 5. Future Work

In our future work, we aim to address several limitations and potential issues identified in this study. First, we plan to expand the diversity and representativeness of the datasets by incorporating real-world weather-affected satellite imagery and data from a wider range of geographic regions in order to improve model robustness and generalization. Additionally, we will explore lightweight variants of the Dense-TNT model to optimize computational efficiency, enabling deployment on resource-constrained platforms such as nanosatellites and embedded systems.

## 6. Conclusions

This paper proposes a novel classification neural network called Dense-TNT for recognizing vehicle types based on satellite remote sensing imagery. Dense-TNT combines a DenseNet layer and TNT layer to capture both local and global information from input images. Our experimental results show that Dense-TNT achieves better recognition performance than other widely used methods, especially under complex weather conditions. Our experiments were designed to validate the feasibility of Dense-TNT based on real-world remote sensing datasets collected from three different regions and containing multiple environment states. Data preprocessing on the datasets was conducted to imitate foggy weather conditions and darkness at light, medium, and heavy levels. The experimental results show that Dense-TNT achieves better recognition performance than the baseline PoolFormer and ViT algorithms, achieving an improvement in accuracy of around 5–10%. Under the foggy weather and darkness conditions, this improvement is even larger. In addition, the proposed model proved effective in a real-world multiclass classification scenario. To summarize, our experiments verify the superior vehicle type classification performance of the proposed Dense-TNT framework under comprehensive weather conditions using remote sensing imagery.

## Figures and Tables

**Table 1:** Model parameter settings.

Model	Number of Layers (L)	Hidden Size (D)	Attention Heads (H)	MLP Size
Dense-TNT s12	12	384	8	-
Dense-TNT s24	24	512	8	-
PoolFormer s12	12	384	-	-
PoolFormer s24	24	512	-	-
Vision Transformer (ViT-L/12)	12	1024	16	4096
Vision Transformer (ViT-L/2)	2	1024	16	4096

**Table 2:** Details of the three datasets. The first column refers to the three different areas where images were taken, the second column refers to the total number of images in the area, and the other two columns refer to the number of sedans and pickups in the dataset, respectively.

Locations	Total Number	Number of Sedans	Number of Pickups
Columbus Ohio	7465	6917	548
Selwyn	4525	3548	1067
Toronto	45,994	44,208	1789

**Table 3:** Example pictures of sedans and pickups. The first column shows four example pictures of sedans and the second column shows four example pictures of sedans.

Sedan	Pickup
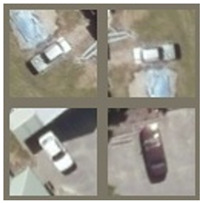	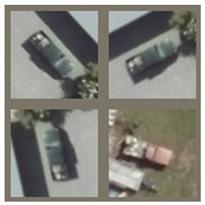

**Table 4:** Experimental results showing the classification accuracy of the models on the three datasets.

	Criteria	Selwyn				Columbus Ohio				Toronto			
Models		**Accuracy**	**Precision**	**Recall**	**F1-Score**	**Accuracy**	**Precision**	**Recall**	**F1-Score**	**Accuracy**	**Precision**	**Recall**	**F1-Score**
Dense-TNT s24		**0.8065**	**0.8211**	0.9558	**0.8810**	**0.7685**	**0.7876**	0.9516	0.8582	**0.8009**	0.8205	**0.9365**	**0.8734**
Dense-TNT s12		0.7971	0.8183	0.9399	0.8722	0.7459	0.7855	0.9109	0.8377	0.7968	**0.8389**	0.9062	0.8706
PoolFormer s24		0.7819	0.7956	**0.9559**	0.8672	0.7675	0.7835	**0.9691**	**0.8634**	0.7584	0.7871	0.9183	0.8469
PoolFormer s12		0.7724	0.7977	0.9424	0.8619	0.7507	0.7812	0.9661	0.8431	0.7456	0.7509	0.9254	0.8441
ViT l12		0.7462	0.7462	0.9256	0.8252	0.7392	0.7421	0.9543	0.8455	0.7300	0.7349	0.9326	0.8401
ViT l2		0.7624	0.7659	0.9435	0.8623	0.7460	0.7486	0.9339	0.8504	0.7510	0.7559	0.9273	0.8560

**Table 5:** Multiclass classification results, showing the classification performance of the six models on the VAID dataset in terms of accuracy, precision, recall, and F1-score.

	Criteria	Accuracy	Precision	Recall	F1-Score
Models					
**Dense-TNT s24**		**0.8753**	**0.8764**	0.9621	**0.9173**
**Dense-TNT s12**		0.8579	0.8657	0.9591	0.9100
**PoolFormer s24**		0.8619	0.8636	**0.9679**	0.9083
**PoolFormer s12**		0.8376	0.8401	0.9484	0.8910
**ViT l12**		0.8229	0.8377	0.9580	0.8938
**ViT l2**		0.8304	0.8459	0.9441	0.8923

**Table 6:** Experimental images under different weather conditions. The four columns respectively refer to images taken under normal weather conditions, light fog conditions, medium fog conditions, and heavy fog conditions.

Origin	Light	Medium	Heavy
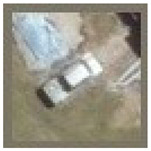	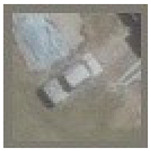	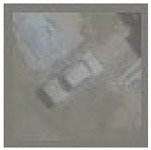	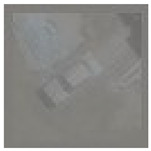

**Table 7:** Results of experiment with image data affected by fog. The first column of the table refers to the different models, while the other columns show the accuracy of the six models in normal weather, light fog, medium fog, and heavy fog, respectively.

	Criteria	Light-Foggy (fog = 0.08)				Medium-Foggy (fog = 0.16)				Heavy-Foggy (fog = 0.24)			
Models		**Accuracy**	**Precision**	**Recall**	**F1-Score**	**Accuracy**	**Precision**	**Recall**	**F1-Score**	**Accuracy**	**Precision**	**Recall**	**F1-Score**
Dense-TNT s24		**0.7941**	0.8240	0.9215	**0.8682**	**0.7961**	**0.7934**	0.9352	**0.8712**	**0.7692**	0.7660	0.9440	**0.8787**
Dense-TNT s12		0.7907	**0.8244**	0.9178	0.8671	0.7839	0.7815	0.9510	0.8382	0.7648	**0.7748**	0.9497	0.8715
PoolFormer s24		0.7665	0.7912	0.9243	0.8490	0.7590	0.7630	0.9594	0.8608	0.7535	0.7663	0.9543	0.8635
PoolFormer s12		0.7631	0.7630	0.9289	0.8641	0.7500	0.7469	**0.9624**	0.8543	0.7371	0.7370	0.9601	0.8469
ViT l12		0.7533	0.7539	**0.9310**	0.8569	0.7456	0.7369	0.9449	0.8431	0.7428	0.7400	0.9627	0.8512
ViT l2		0.7566	0.7495	0.7297	0.8585	0.7394	0.7402	0.9573	0.8482	0.7383	0.7369	**0.9659**	0.8471

**Table 8:** Experimentalimages showing different darkness conditions. The four columns respectively refer to images under normal conditions and under light, medium, and heavy darkness conditions.

Origin	Light	Medium	Heavy
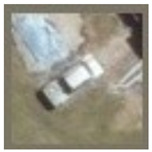	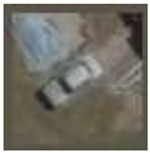	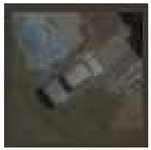	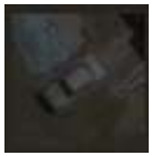

**Table 9:** Results of experiments with data affected by darkness. The first column of the table refers to the six models, while the other columns show the accuracy of the models under normal conditions and light, medium, and heavy darkness conditions.

	Criteria	Light-Darkness (θ=0.8)				Medium-Darkness (θ=0.64)				Heavy-Darkness (θ=0.32)			
Models		**Accuracy**	**Precision**	**Recall**	**F1-Score**	**Accuracy**	**Precision**	**Recall**	**F1-Score**	**Accuracy**	**Precision**	**Recall**	**F1-Score**
Dense-TNT s24		**0.8105**	0.8122	0.8981	**0.8912**	**0.8044**	**0.8137**	0.9152	**0.9048**	**0.7797**	0.7561	0.9259	**0.8761**
Dense-TNT s12		0.7852	**0.8341**	0.9062	0.8783	0.7618	0.7795	0.9150	0.8283	0.7486	**0.7847**	0.9053	0.8517
PoolFormer s24		0.7432	0.7902	0.9142	0.8370	0.7590	0.7541	0.9495	0.8267	0.7535	0.7569	0.9435	0.8295
PoolFormer s12		0.7631	0.7750	0.9156	0.8566	0.7500	0.7291	**0.9604**	0.8534	0.7199	0.7257	0.9534	0.8225
ViT112		0.7533	0.7129	**0.9317**	0.8776	0.7528	0.7274	0.9449	0.8264	0.7244	0.7390	0.9567	0.8591
ViT12		0.7566	0.7481	0.7957	0.8585	0.7394	0.7337	0.9601	0.8385	0.7223	0.7469	**0.9660**	0.8351

## Data Availability

The dataset used in this paper can be found at: http://gdo-datasci.ucllnl.org/cowc/ (accessed on 12 September 2024) and https://vision.ee.ccu.edu.tw/aerialimage/ (accessed on 22 November 2024).

## References

[1] Luo R., Zhang Y., Zhou Y., Chen H., Yang L., Yang J., Su R. Deep Learning Approach for Long-Term Prediction of Electric Vehicle (EV) Charging Station Availability. Proceedings of the 2021 IEEE International Intelligent Transportation Systems Conference (ITSC).

[2] Luo R., Song Y., Huang L., Zhang Y., Su R. (2022). AST-GIN: Attribute-Augmented Spatial-Temporal Graph Informer Network for Electric Vehicle Charging Station Availability Forecasting. arXiv.

[3] Song Y., Zhao H., Luo R., Huang L., Zhang Y., Su R. (2022). A SUMO Framework for Deep Reinforcement Learning Experiments Solving Electric Vehicle Charging Dispatching Problem. arXiv.

[4] Huang L., Zhao S., Luo R., Su R., Sindhwani M., Chan S.K., Dhinesh G.R. An Incremental Map Matching Approach with Speed Estimation Constraints for High Sampling Rate Vehicle Trajectories. Proceedings of the 2022 IEEE 17th International Conference on Control & Automation (ICCA).

[5] Amato G., Ciampi L., Falchi F., Gennaro C. Counting vehicles with deep learning in onboard uav imagery. Proceedings of the 2019 IEEE Symposium on Computers and Communications (ISCC).

[6] Luo R., Su R. Traffic Signal Transition Time Prediction Based on Aerial Captures during Peak Hours. Proceedings of the 2020 16th International Conference on Control, Automation, Robotics and Vision (ICARCV).

[7] Wang A.X., Tran C., Desai N., Lobell D., Ermon S. Deep transfer learning for crop yield prediction with remote sensing data. Proceedings of the 1st ACM SIGCAS Conference on Computing and Sustainable Societies.

[8] Barmpoutis P., Papaioannou P., Dimitropoulos K., Grammalidis N. (2020). A review on early forest fire detection systems using optical remote sensing. Sensors.

[9] Mahapatra D., Bozorgtabar B., Garnavi R. (2019). Image super-resolution using progressive generative adversarial networks for medical image analysis. Comput. Med. Imaging Graph..

[10] Wu Q., Ren W., Cao X. (2019). Learning interleaved cascade of shrinkage fields for joint image dehazing and denoising. IEEE Trans. Image Process..

[11] Ma X., Grimson W.E.L. Edge-based rich representation for vehicle classification. Proceedings of the Tenth IEEE International Conference on Computer Vision (ICCV’05) Volume 1.

[12] Zhang C., Chen X., Chen W.b. A PCA-based vehicle classification framework. Proceedings of the 22nd International Conference on Data Engineering Workshops (ICDEW’06).

[13] Ji P., Jin L., Li X. Vision-based vehicle type classification using partial Gabor filter bank. Proceedings of the 2007 IEEE International Conference on Automation and Logistics.

[14] Shan Y., Sawhney H.S., Kumar R. (2008). Unsupervised learning of discriminative edge measures for vehicle matching between nonoverlapping cameras. IEEE Trans. Pattern Anal. Mach. Intell..

[15] Jiang M., Li H. (2014). Vehicle classification based on hierarchical support vector machine. Computer Engineering and Networking.

[16] Hsieh J.W., Yu S.H., Chen Y.S., Hu W.F. (2006). Automatic traffic surveillance system for vehicle tracking and classification. IEEE Trans. Intell. Transp. Syst..

[17] Gupte S., Masoud O., Martin R.F., Papanikolopoulos N.P. (2002). Detection and classification of vehicles. IEEE Trans. Intell. Transp. Syst..

[18] Lai A.H., Fung G.S., Yung N.H. Vehicle type classification from visual-based dimension estimation. Proceedings of the ITSC 2001, 2001 IEEE Intelligent Transportation Systems, Proceedings (Cat. No. 01TH8585).

[19] Lowe D.G. (2004). Distinctive image features from scale-invariant keypoints. Int. J. Comput. Vis..

[20] Zhang Z., Tan T., Huang K., Wang Y. (2011). Three-dimensional deformable-model-based localization and recognition of road vehicles. IEEE Trans. Image Process..

[21] Pal T. Visibility enhancement of fog degraded image sequences on SAMEER TU dataset using dark channel strategy. Proceedings of the 2018 9th International Conference on Computing, Communication and Networking Technologies (ICCCNT).

[22] Bera S., Shrivastava V.K. (2020). Analysis of various optimizers on deep convolutional neural network model in the application of hyperspectral remote sensing image classification. Int. J. Remote Sens..

[23] Zhang Q., Zhang M., Chen T., Sun Z., Ma Y., Yu B. (2019). Recent advances in convolutional neural network acceleration. Neurocomputing.

[24] Yang J., Chen H., Xu Y., Shi Z., Luo R., Xie L., Su R. Domain adaptation for degraded remote scene classification. Proceedings of the 2020 16th International Conference on Control, Automation, Robotics and Vision (ICARCV).

[25] Dosovitskiy A., Beyer L., Kolesnikov A., Weissenborn D., Zhai X., Unterthiner T., Dehghani M., Minderer M., Heigold G., Gelly S. (2020). An image is worth 16 × 16 words: Transformers for image recognition at scale. arXiv.

[26] Dong Z., Wu Y., Pei M., Jia Y. (2015). Vehicle type classification using a semisupervised convolutional neural network. IEEE Trans. Intell. Transp. Syst..

[27] Parmar N., Vaswani A., Uszkoreit J., Kaiser L., Shazeer N., Ku A., Tran D. Image transformer. Proceedings of the International Conference on Machine Learning.

[28] Zhao H., Jia J., Koltun V. Exploring self-attention for image recognition. Proceedings of the IEEE/CVF Conference on Computer Vision and Pattern Recognition.

[29] Child R., Gray S., Radford A., Sutskever I. (2019). Generating long sequences with sparse transformers. arXiv.

[30] Yu W., Luo M., Zhou P., Si C., Zhou Y., Wang X., Feng J., Yan S. Metaformer is actually what you need for vision. Proceedings of the IEEE/CVF Conference on Computer Vision and Pattern Recognition.

[31] Han K., Wang Y., Chen H., Chen X., Guo J., Liu Z., Tang Y., Xiao A., Xu C., Xu Y. (2022). A survey on vision transformer. IEEE Trans. Pattern Anal. Mach. Intell..

[32] Han K., Xiao A., Wu E., Guo J., Xu C., Wang Y. (2021). Transformer in transformer. Adv. Neural Inf. Process. Syst..

[33] Liu Z., Lin Y., Cao Y., Hu H., Wei Y., Zhang Z., Lin S., Guo B. Swin transformer: Hierarchical vision transformer using shifted windows. Proceedings of the IEEE/CVF International Conference on Computer Vision.

[34] Chen C.F., Panda R., Fan Q. (2021). Regionvit: Regional-to-local attention for vision transformers. arXiv.

[35] Chu X., Tian Z., Wang Y., Zhang B., Ren H., Wei X., Xia H., Shen C. (2021). Twins: Revisiting the design of spatial attention in vision transformers. Adv. Neural Inf. Process. Syst..

[36] Lin H., Cheng X., Wu X., Yang F., Shen D., Wang Z., Song Q., Yuan W. (2021). Cat: Cross attention in vision transformer. arXiv.

[37] Zhou D., Kang B., Jin X., Yang L., Lian X., Jiang Z., Hou Q., Feng J. (2021). Deepvit: Towards deeper vision transformer. arXiv.

[38] Chu X., Tian Z., Zhang B., Wang X., Wei X., Xia H., Shen C. (2021). Conditional positional encodings for vision transformers. arXiv.

[39] Wu K., Peng H., Chen M., Fu J., Chao H. Rethinking and improving relative position encoding for vision transformer. Proceedings of the IEEE/CVF International Conference on Computer Vision.

[40] Touvron H., Cord M., Sablayrolles A., Synnaeve G., Jégou H. Going deeper with image transformers. Proceedings of the IEEE/CVF International Conference on Computer Vision.

[41] Adjei-Mensah I., Zhang X., Baffour A.A., Agyemang I.O., Yussif S.B., Agbley B.L.Y., Sey C. Investigating Vision Transformer Models for Low-Resolution Medical Image Recognition. Proceedings of the 2021 18th International Computer Conference on Wavelet Active Media Technology and Information Processing (ICCWAMTIP).

[42] Luo R. (2019). Mission Design for the VELOX-Lyon-1 (Rebranded SCOOBI) Student CubeSat.

[43] She X., Zhang D. Text classification based on hybrid CNN-LSTM hybrid model. Proceedings of the 2018 11th International Symposium on Computational Intelligence and Design (ISCID).

[44] Huang G., Liu Z., Van Der Maaten L., Weinberger K.Q. Densely connected convolutional networks. Proceedings of the IEEE Conference on Computer Vision and Pattern Recognition.

[45] Mundhenk T.N., Konjevod G., Sakla W.A., Boakye K. (2016). A Large Contextual Dataset for Classification, Detection and Counting of Cars with Deep Learning. arXiv.

[46] Lin H.Y., Tu K.C., Li C.Y. (2020). VAID: An Aerial Image Dataset for Vehicle Detection and Classification. IEEE Access.

